# Supplementary material for: Implementation of clinically relevant and robust fMRI-based language lateralization: Choosing the laterality index calculation method
Source: PLoS One. 2020 Mar 12;15(3):e0230129. doi: 10.1371/journal.pone.0230129 (PMC7067428; doi:10.1371/journal.pone.0230129)
Supplement: Attachment — Submitted filename: ResponseToReviewers_final_R1.docx [file pone.0230129.s003.docx]

**Response to Reviewers for Manuscript “Implementation of clinically relevant and robust fMRI-based language lateralization: choosing the laterality index calculation method”, PONE-D-19-25144**

***17 December 2019***

**Reviewer #1:**

**Thank you for a well-written article on an interesting topic, keeping the useability of the results for clinicians and researchers in mind throughout the paper. I have some minor comments.**

We thank Reviewer #1 for the useful suggestions; we believe addressing these has made the manuscript clearer.

**R1.1** **I cannot find the full dataset (fMRI raw data or statistical parameter maps) provided anywhere as attachment, supplementary data or link to data repository.**

Subjects were recruited, and informed written consent was obtained, with ethical approval from the UK National Research Ethics Service (REC 04/Q0706/72). Under the UK Health Research Authority (HRA) system the Research Ethics Committee do not directly regulate access to data, and should not be approached about such issues. Data access lies under the overall governance of the sponsoring organisation - and ultimately depends on what the subjects have consented to. The information sheet given to the healthy volunteers participating to this study states ‘We may share the images we collect with others involved in the research (including researchers at other hospitals or universities, or working for the funding bodies)’. Although we may be able to ask for this in future studies, for the data reported here we do not have the consent from the participants to a) upload the images to a repository b) make the images publicly available c) share the images with researches not involved in this research (and we do not have permission to recontact the participants about this). Therefore, unfortunately, the fMRI raw images and the calculated images (statistical parametric maps) cannot be provided on this occasion.

Nonetheless, we do completely appreciate the importance of making all the raw numerical data used in the paper available. We have therefore provided all LI values calculated for each subject using the four presented methods, and also the resulting subject rankings, as supplementary data (S1 Table). We have also provided the curveLI ‘mean’ and ‘95% confidence interval’ curves for all tasks and ROIs shown in Figure 4 as data points (supplementary data S2 Table). These define and summarize our reference cohort, and can be used for future single subject or cohort comparisons. All these raw data constitute the results from this research

**R1.2** **Line 168/169 on page 8 would read better if the numbers and letters referred to were put in quotation marks, e.g. "... with 'a' designating Broca's area"**

We have adopted the suggested reformatting in the revised manuscript.

**R1.3** **The description of the novel method, AUCLI, was lacking some details that would aid reproducibility of the method. First, line 213-215 reads "... count the number of voxels with values above the threshold for all t-values present in the ROIs". Is this meant to apply to each threshold present in the ROIs? The sentence reads as if one threshold is set, but this cannot be the case as the manuscript is advocating the use of threshold-independent LIs.**

Yes, this is meant to apply to all thresholds (t-values) present in the ROIs. Each t-value present in the ROIs is used as a threshold for which the number of voxels with value above the threshold is calculated, forming a cumulative histogram.

To make this clearer, we have modified the description of the method as follows (page 10 line 213-227):

Another way of looking at the difference in activation in left and right ROI is to consider the number of voxels with values above the threshold for all possible thresholds [42]. This is achieved by setting every t-value present in the ROIs as a threshold and record the number of voxels with value above the threshold. The cumulative histograms histograms of the obtained number of voxel vs threshold for left and right ROI (shown in Fig 2), can then be compared. The new method we propose in this work calculates the areas under these histograms and quantitatively compares them using the following formula:

$AUCLI=(AUCL-AUCR)/(AUCL+AUCR)$	(6)

where AUCL and AUCR are the areas under the cumulative histogram calculated for the left and right ROI, respectively. The areas under the cumulative histograms were calculated using the trapezoidal numerical integration available as a function in MATLAB.

**R1.4** **The first sentence of the Results, at page 11 line 239 refers to LI versus threshold plots that are likely referring to the conventional LI. Please do state this more clearly in this sentence. As LI is not consistently used only to refer to the conventional LI calculation, it is helpful to be very consistent in naming. Likewise, in the description of Fig 4 it is helpful to refer to the in-manuscript name of the method; curveLI.**

We have adopted the suggested renaming/clarification in the revised manuscript.

**R1.5** **In the figure text of Fig 5 (page 13 lines 295-296) there's a reference to outliers. The manuscript is lacking a clear description of outlier detection chosen, and if there is need for outlier detection (rationale, implications).**

In this case, with ‘outliers’ we are just referring to single subject LI values which fall outside the distribution represented by the boxplots in Fig 5. We have included them in these graphs just to give a more truthful representation of the distribution of values.

We have now added an explanation of how the outliers are defined in the caption of Fig 5 (page 13 line 298-303)

**Fig 5: Boxplots showing the variation between subjects within each method.**

For each box, the central line indicates the median. The bottom and top edges indicate the first quartile q_1_ and third quartile q_3_, respectively. The whiskers extend to the most extreme data points not considered as outliers. Outliers are shown individually as ‘+’ on the plots. Outliers are hereby defined as values larger than q_3_+w(q_3_-q_1_) or smaller than q_1_-w(q_3_-q_1_), where w is the maximum whisker length.

**R1.6** **At page 17/18 there is not a clear positive answer to whether other methods than CurveLI adhere to criterion 3 for suitability for clinical implementation: "allow an easy subject comparison". Therefore, the conclusion drawn in line 412/413 "Taking these five criteria into account, the novel method proposed in this work can be considered suitable for the clinical routine" cannot be drawn which undermines one of the main findings of the manuscript. Of course, the authors do show means of comparing between subjects throughout the manuscript, so this should be described in this section together with a judgment of whether this is adequate enough to be deemed as "easy", or else rephrase their conclusions regarding clinical implementability.**

We understand the need for clarification here. From a radiologist’s point of view, the simpler the information the better - and therefore a single summarizing numerical value is convenient for understanding and comparing results from different subjects. As all presented methods can provide a summarizing LI value, they all satisfy criterion 3) in our opinion. The curveLI method just has the additional advantage of offering a visual comparison while retaining the more complex information of the variation of LI with number of activated voxels. A clinical user would appreciate the visual information, but its meaning would need to be explained.

We have clarified this in the Discussion (page 18, line 406-419):

All presented threshold-independent methods can provide a single summarizing LI value, which makes comparison between subjects easy, therefore satisfying criterion 3). However, the *curveLI* method also offers visual comparison of curves plotted in a single graph (Fig 4).Such a comparison is valid only if the number of activated voxels is appropriately normalized (for example to the total number of voxels with positive values within the SPM for each subject, as done in this work), or if the total number of positive voxels within the ROI considered is the same for all subjects, as done by Abbott and colleagues [24]. When a reference cohort is available, the curveLI method offers an easy visual comparison (direct assessment of where the patient curve lies in respect to the mean LI curve and 95% confidence interval), while retaining the more complex information of the smooth dependence of the the LI on the total number of activated voxels.

**R1.7** I**t should be noted that the colors and gradient used in Fig 6 are virtually indiscriminable when viewed as a black and white printout, it would be advisable to switch to colors with a clear difference in b/w.**

The plots in Figure 6 were designed to have a good visual representation in colour as this should be always available online in PLOS ONE.

We believe the figure works best in color; therefore we have not produced an alternative black and white figure. Nevertheless, we have added all LI values used to produce the plots as supplementary data (S1 Table).

**Reviewer #2:**

**The authors present a study comparing methods for calculating laterality index. While the findings may not have broad appeal, they will certainly be of use for groups performing clinical fMRI for pre-surgical mapping.

I have a number of comments, though.**


**R2.1.** **Why was such a slow TR of 3 seconds chosen? It has been apparent for a while that the sensitivity of fMRI increases with decreasing TR.**

We thank Reviewer #2 for all the comments. This study was performed in a clinical scanner at 1.5 T, and our implementation reflects what is achievable in this setting. The sequence parameters used for this study, and particularly the TR, resulted from a compromise between SNR and achieving the required brain coverage with a spatial resolution of 2.5x2.5x3 mm^3^. With these conditions, the sequence would not actually allow a shorter TR; to allow a TR of 2 seconds or less, we would have had to reduce the spatial resolution to 4x4x4 mm^3^.

**R2.2. When using SPM, it is generally recommended to choose a smoothing kernel that is approximately 2x the size of the acquired voxel (see Friston et al., 1996 NeuroImage; Ball et al., 2012 Human Brain Mapping; Pajula and Tohka 2014, MRI; Liu et al., 2017, J Neuroscience Methods). Given that the acquired voxel is 2.5x2.5x3mm, can the authors justify using an 8mm smoothing kernel, which is almost 3x the size of the acquired voxel?**

Several reports on task-based language fMRI (e.g. Abbott et al 2010, whose method we implemented in our study, or Zhang et al 2015 <https://www.nature.com/articles/srep11704>) use an isotropic smoothing kernel of 8mm with a base resolution of 3x3x3mm^3^, and were acquired at 3T. Our data was acquired at 1.5T and we deemed an 8mm smoothing kernel to be an appropriate choice and it is also the default in SPM12.

**R2.3. What did the authors include in their GLM beyond the task regressors? Were the realignment parameters estimated during the realignment step included as covariates of no interest? What steps were taken to mitigate the effect of noise in the data? What quality checks were performed on the MRI data? Individual examination for excessive head motion?**

Using motion as a regressor in the GLM has been shown to improve reproducibility in research fMRI studies but is not a technique typically applied in strictly clinical fMRI tools for neurosurgical planning. In this work we decided therefore to not apply this technique and to instead maintain data quality through ensuring motion was kept to minimum.

The effect of noise in the data was not the main focus of this work and was not investigated.

The fMRI data was visually inspected for gross head motion and artefact during the time series and the motion regressor plot was inspected for significant motion. The registration of the fMRI data with the structural image volume was also visually checked.

We kept the data pre-processing steps as standard as possible as we believe this is what is typically achievable in a clinical (rather than a research) setting.

**R2.4. I do not understand the purpose of Figure 6. What is it showing that could not be shown in Figure 5, if the authors included the individual data points as part of the box and whisker plots in Figure 5? The authors need to better explain what is being shown in Figure 6, especially why it is important to color code the subjects based on curveLI method?**

Figure 6 shows the subject rankings obtained with the different methods, which is what is compared with the Spearman’s correlation coefficients. The subject ranking could only be made visible in the boxplots of Figure 5 if each data point was labelled with the corresponding subject number. This would however result in ‘crowded’ boxplots, which, in our opinion, would impede the visualisation of the overall distribution, which is the main purpose of Figure 5. The choice of basing the ranking representation on the curveLI method is arbitrary, any of the method could have been chosen as reference. Figure 6 is particularly effective in highlighting differences in rankings as these are visualized as crossing lines in the plots.

This has been clarified in the manuscript by improving the description of Figure 6 in the caption as follows (page 13, line 304-311):

**Fig 6: Visual comparison of the subject rankings obtained with the different methods.**

Within each graph, a gradual color change from blue for low LI values to red for high LI values is attributed to each subject, reflecting the subject ranking obtained with the curveLI method (left side of the plot). Crossing lines indicate differences in subject rankings between the methods.

**R2.5. Did the authors consider acquiring multiple runs of each task in a single session or performing multiple scanning sessions to enable any sort of test-retest or cross-validation of the results? This would be an interesting result to see in terms of how robust each LI method is.**

This would indeed have been interesting. However, the acquisition time for each subject was already over 40 minutes, including all the other sequences, and an extension might have introduced differences in performance due to a decrease in attention from the subject. We apply similar criteria for the total length of the exam for clinical subjects, as data quality deteriorates in lengthy and demanding acquisitions. In this case, our primary objective was the creation of a validation cohort also useful for the clinical implementation of fMRI-based language lateralization. We had to consider the generous but limited hospital resources in terms of scanner availability for development – which would also have prevented repeating the acquisition over multiple sessions.

**R2.6. In the conclusions, the authors mention that laterality index measures can be influenced by post-processing method. Given the results presented in the manuscript, how did the authors arrive at this conclusion? I did not see any indication that the authors varied anything but whether LI was calculated at the hemispheric level or using language-related ROIs. To justify this conclusion, I would have expected to see the authors comparing smoothing kernels, standard software packages/pipelines, the inclusion/exclusion of noise confounds in the GLM, etc.**

The post-processing method mentioned in the conclusion only refers to the LI calculation method used in the post-processing as this is the aspect we considered in this work. If different studies uses different methods to calculate a summarizing LI value, can these values really be compared? This is the question the manuscript addresses and while we found good agreement between the subject rankings, the actual LI values are not identical for each subject with the four methods we have used. So even when the same task(s) and the same fMRI data analysis are used to obtain the activation maps, the resulting LIs might still differ just because of a different LI calculation.

To remove the ambiguity, we have clarified the sentence in the conclusion (page 22, line 507-509):

Our results highlight that the laterality index is not an absolute measure, as numerous factors - some purely related to the LI calculation method - can influence its value.

**R2.7. The authors have omitted many of the required references and acknowledgments for using the Harvard Oxford and Julich Histological atlases (https://fsl.fmrib.ox.ac.uk/fsl/fslwiki/Atlases).**

The missing references listed for the Harvard Oxford and Jülich Hsitological Atlases on the mentioned website were added to the list of references in the manuscript as references 34-40. For the Jülich histological Atlas, we also kept reference 41, which corresponds to the paper listed for Broca’s region as this is the specific structure used in this work (<https://fsl.fmrib.ox.ac.uk/fsl/fslwiki/Atlases/Juelich>).
